# Supplementary material for: Robust induction of B cell and T cell responses by a third dose of inactivated SARS-CoV-2 vaccine
Source: Cell Discov. 2022 Feb 1;8:10. doi: 10.1038/s41421-022-00373-7 (PMC8803973; doi:10.1038/s41421-022-00373-7)
Supplement: Supplementary file 1 — Supplementary Information [file 41421_2022_373_MOESM1_ESM.pdf]

Supplementary information

Supplementary Table S1. Demographics and vaccination-related adverse events

		Morning group	Afternoon group	Total
Participants, N		25	25	50
Age, mean (SD)		26.88 (4.86)	27.88 (6.93)	27.38 (5.94)
Gender, male (%)		9 (36.0)	11 (44.0)	26 (41.3)
Adverse events,	N (%)	5 (20.0%)	1 (4.0%)	6 (12.0%)
Injection site symptoms	Induration, N (%)	1 (4.0%)	0 (0.0%)	1 (2.0%)
	Swollen, N (%)	1 (4.0%)	0 (0.0%)	1 (2.0%)
Systematic symptoms	Fever, N (%)	2 (8.0%)	0 (0.0%)	2 (4.0%)
	Fatigue, N (%)	2 (8.0%)	1 (4.0%)	3 (6.0%)
Respiratory symptoms	Runny nose, N (%)	1 (4.0%)	0 (0.0%)	1 (2.0%)
	Cough, N (%)	1 (4.0%)	0 (0.0%)	1 (2.0%)
	Sore throat, N (%)	1 (4.0%)	0 (0.0%)	1 (2.0%)

Supplementary Table S2. Demographics of the COVID-19 convalescent patients

Patient ID	Age range	Gender	Days post-diagnosis	NAbs* (AU/ml)
#1	21-25	F	407	28.67
#2	36-40	F	406	582.92
#3	31-35	F	411	14.79
#4	36-40	M	409	74.09
#5	46-50	F	403	23.55

*NAbs: neutralizing antibodies

Supplementary Table S3. Antibodies used in this study.

Marker	Fluorophore	Vendor	Cat#	Clone
CD4	BV510	Biolegend	357420	A161A1
CD8	Percp	Biolegend	344708	SK1
CD45RA	BV650	Biolegend	304136	HI100
CD3	Pacific Blue™	Biolegend	300330	HIT3a
CD137	PE	Biolegend	309804	4B4-1
OX40	BV605	Biolegend	350028	ACT35
CD69	Super Bright 436	Invitrogen	62-0699-42	FN50
CCR7	APC/cy7	Biolegend	353212	G043H7
CD19	PE/cy7	Biolegend	302216	HIB19
PD-1	PE/cy7	Biolegend	367414	NAT105
CXCR5	BV711	Biolegend	356934	J252D4
CD27	AF700	Biolegend	356416	M-T271
IgD	FITC	Biolegend	348206	IA6-2
CD38	BV650	Biolegend	356620	HB-7
IgM	BV605	Biolegend	314524	MHM-88
IgG	AF647	Biolegend	302216	HIB19
IgA	PE	Biolegend	333504	HM47
IFNγ	PE-CY7	Biolegend	502528	4S.B3
Zombie	Zombie Red	Biolegend	423110	——
Streptavidin	BV421	Biolegend	405226	——
Streptavidin	BV510	Biolegend	405233	——
Streptavidin	BV785	Biolegend	405249	——

Supplementary Figures and legends

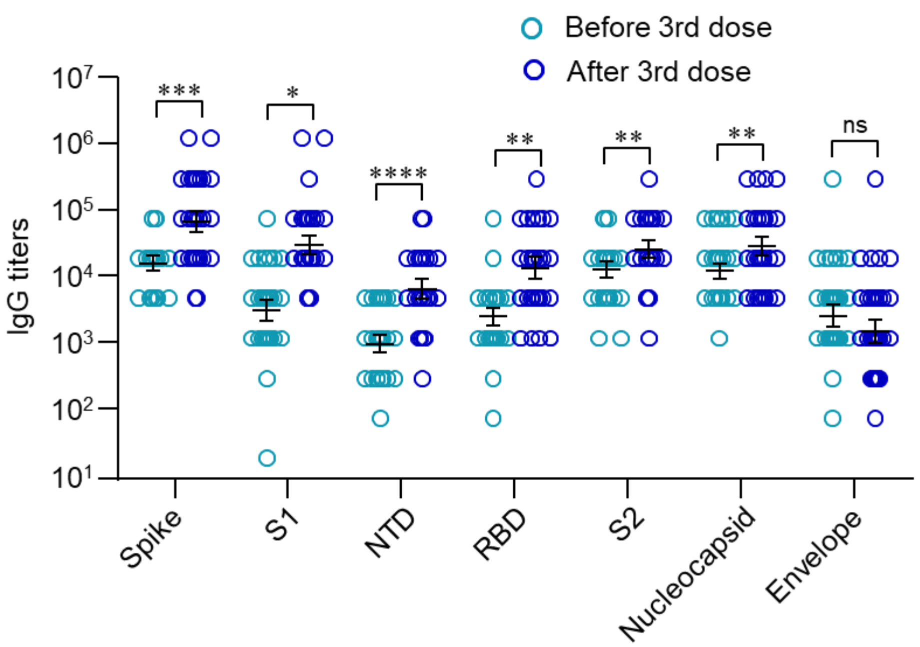

Supplementary Fig. S1 Antibody spectrum induced by inactivated vaccine BBIBP-CorV. Study was carried out as in Fig.1. IgG against SARS-CoV-2 spike, spike S1, spike N terminal domain (NTD), receptor-binding domain (RBD), spike S2, nucleocapsid and envelope protein before (d180) and after (d194) the third dose were measured by ELISA. ns, not significant, **P*<0.05, ***P*<0.01, ****P*<0.001 and *****P*<0.0001 by paired Student’s t test.

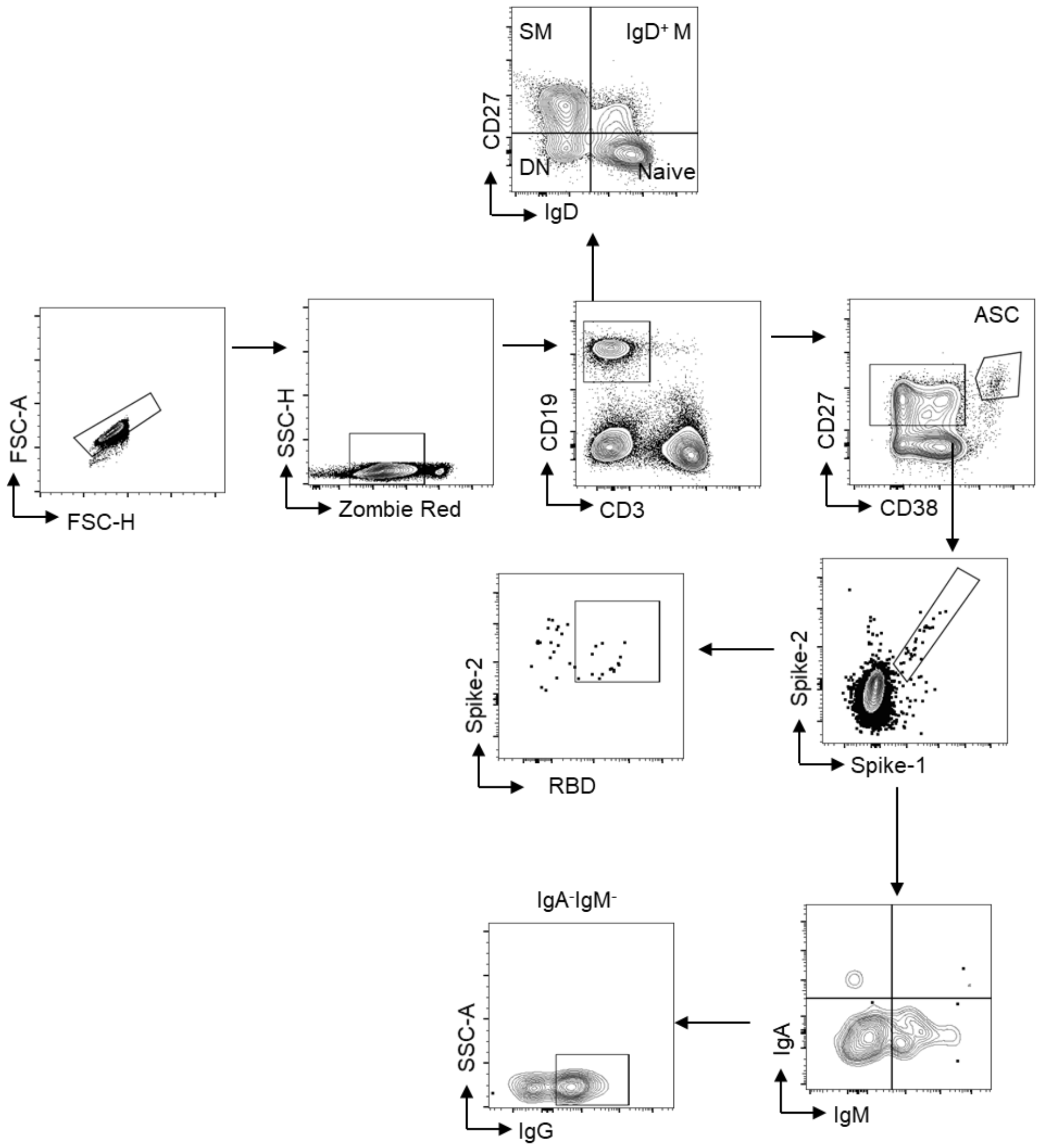

Supplementary Fig. S2 Gating strategy for B cell analysis. PBMCs were collected on day (d) 180 before the third dose and d187 after the third dose of vaccination. Cells were stained with antibodies against CD3, CD19, CD27, IgD, IgM, IgG, IgA and CD38. Dead cells were first gated out by gating on Zombie Red$^-$ population. Cells were then gated on CD19$^+$ B cells for further analysis: naïve B cels: IgD$^+$CD27$^-$, IgD$^+$ memory B cells: IgD$^+$CD27$^+$, switched memory B cells: IgD$^-$CD27$^+$, double-negative (DN) B cells: IgD$^-$CD27$^-$, antibody-secreting cells (ASC): CD38$^+$CD27$^+$.

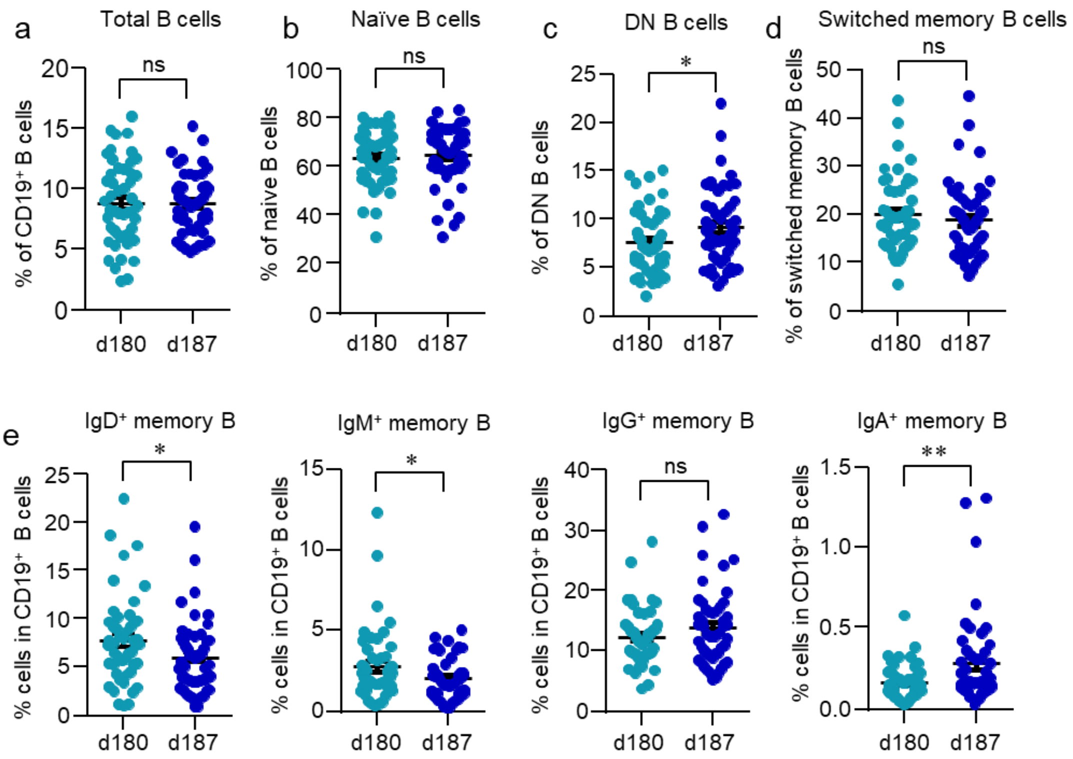

Supplementary Fig. S3 B cell response to a third dose of inactivated SARS-CoV-2 vaccine. Blood samples were collected on day (d) 180 and d187. Data were acquired by flow cytometry. The gating strategy is shown in Supplementary Fig. S2. B cell subsets were summarized and shown as dot plots. Data are expressed meat ± SEM. ns, not significant, *P<0.05, **P<0.01. Comparisons were done by Student's t test.

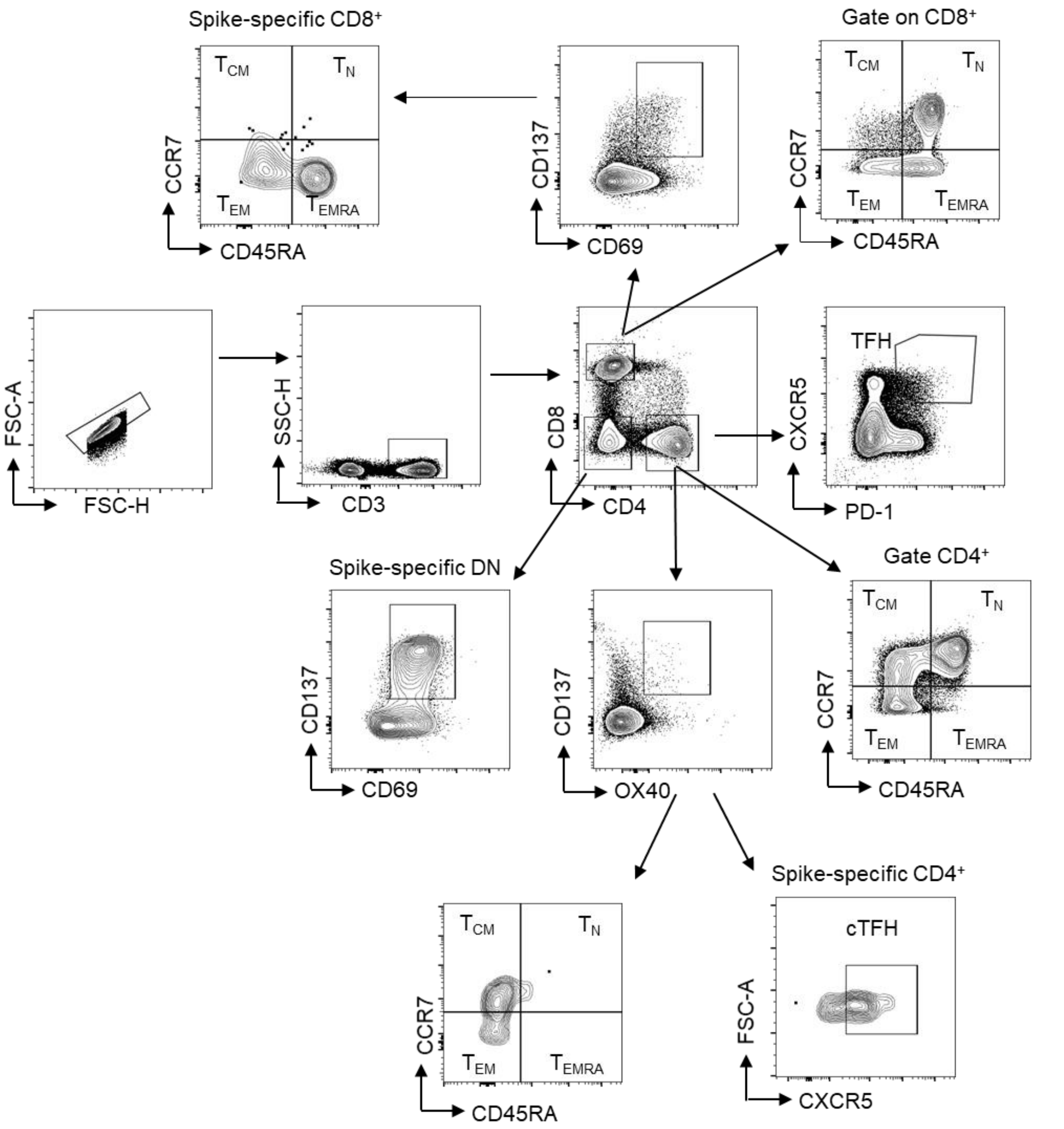

Supplementary Fig. S4 Gating strategy for T cell analysis. PBMCs were collected on day (d) 180 before the third dose and d187 after the third dose vaccination. Cells were stained with antibodies against CD3, CD4, CD8, IgD, IgM, IgG, IgA and CD38. T cell phenotyping: TFH cells: $CD4^+CXCR5^+PD\text{-}1^+$, Spike-specific $CD4^+$ T cells: $OX40^+$4-$1BB^+$, Spike-specific $CD8^+$ T cells: $CD69^+$4-$1BB^+$, naïve T cells (T_N): $CD45RA^+CCR7^+$, effector memory T cells (T_{EM}): $CD45RA^-CCR7^-$, central memory T cells (T_{CM}): $CD45RA^-CCR7^+$, terminally differentiated T cells (T_{EMRA}): $CD45RA^+CCR7^-$.

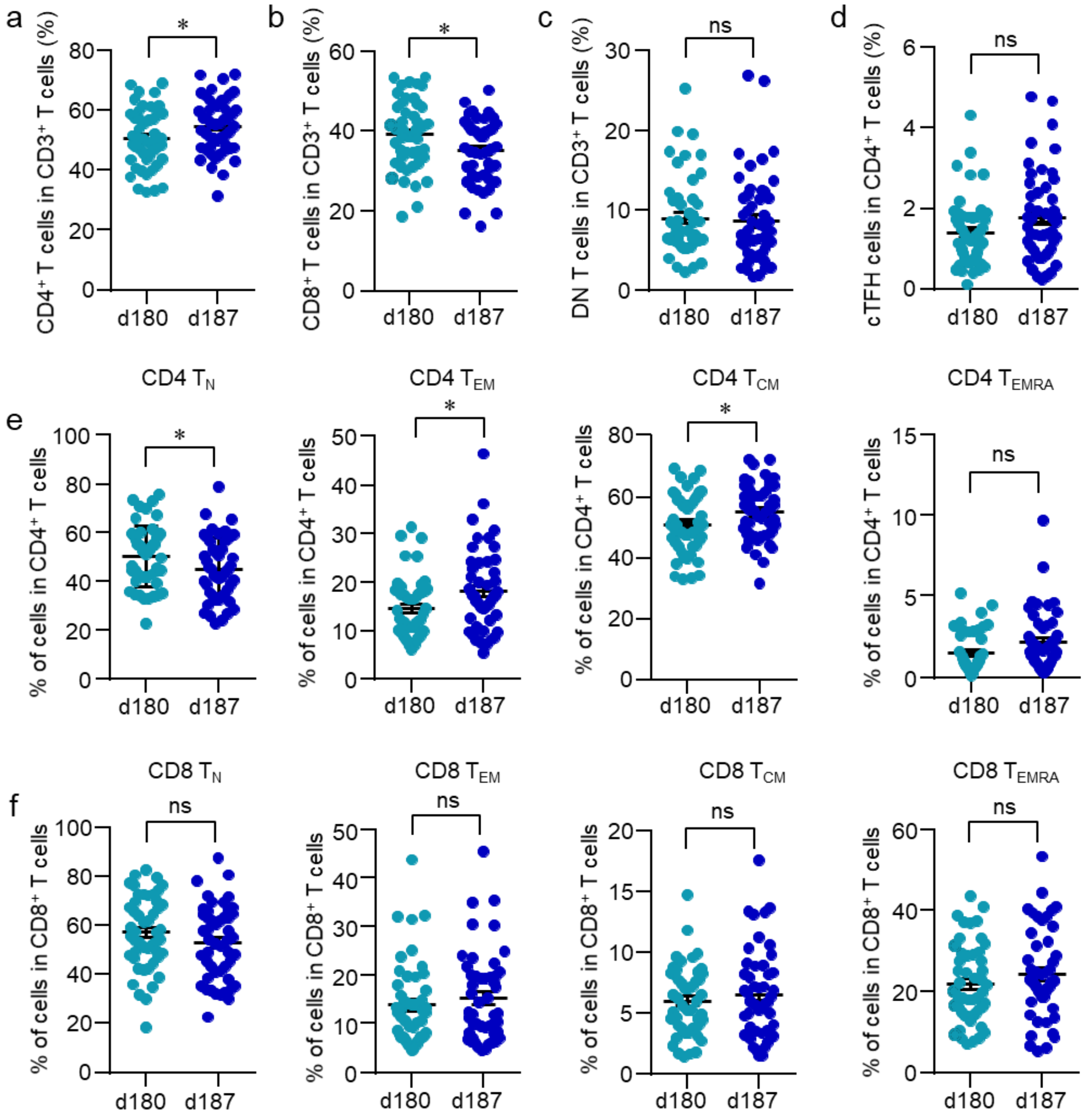

Supplementary Fig. S5 T cell response to a third dose of inactivated SARS-CoV-2 vaccine. Blood samples were collected on day (d) 180 and d187. Data were acquired by flow cytometry. The gating strategy is shown in Supplementary Fig. S4. T cell subsets were summarized and shown as dot plots. Data are expressed mean ± SEM. ns, not significant, **P*<0.05, Comparisons were done by Student's t test.

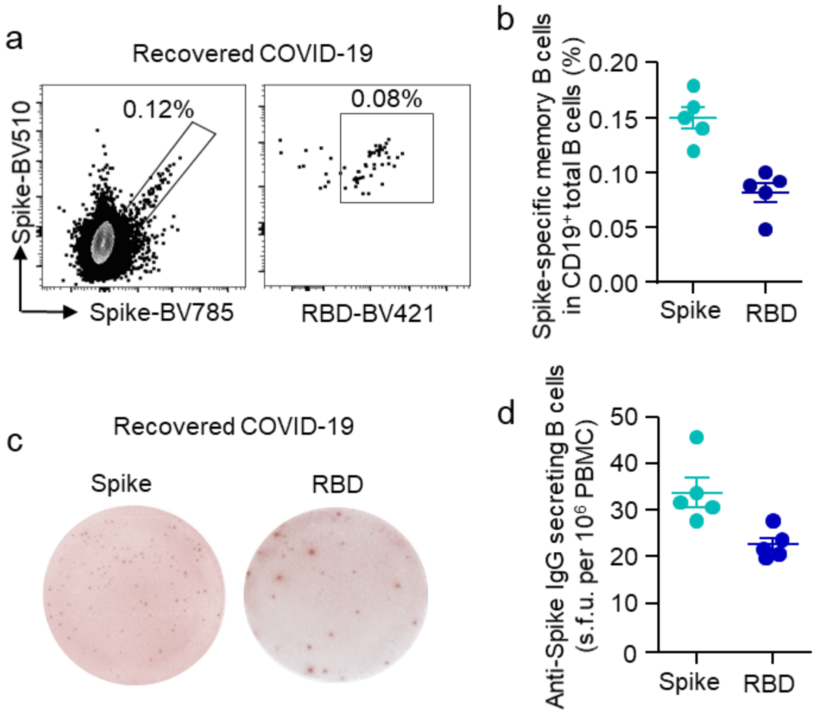

Supplementary Fig. S6 SARS-CoV-2-specific memory B cells in COVID-19 convalescent patients. PBMCs were isolated from patients who have recovered from COVID-19 over 13 months. (a, b) Spike- and receptor-binding domain (RBD)-specific memory B cells were measured by flow cytometry. Representative FACS plots showing spike- and RBD-specific memory B cells. Data were summarized from 5 independent samples. (c, d) SARS-CoV-2-specific memory B cells were further detected by ELISpot. Images of ELISpot wells were shown. Spot-forming units (s.f.u) were summarized and adjusted to 10^6 PBMCs.

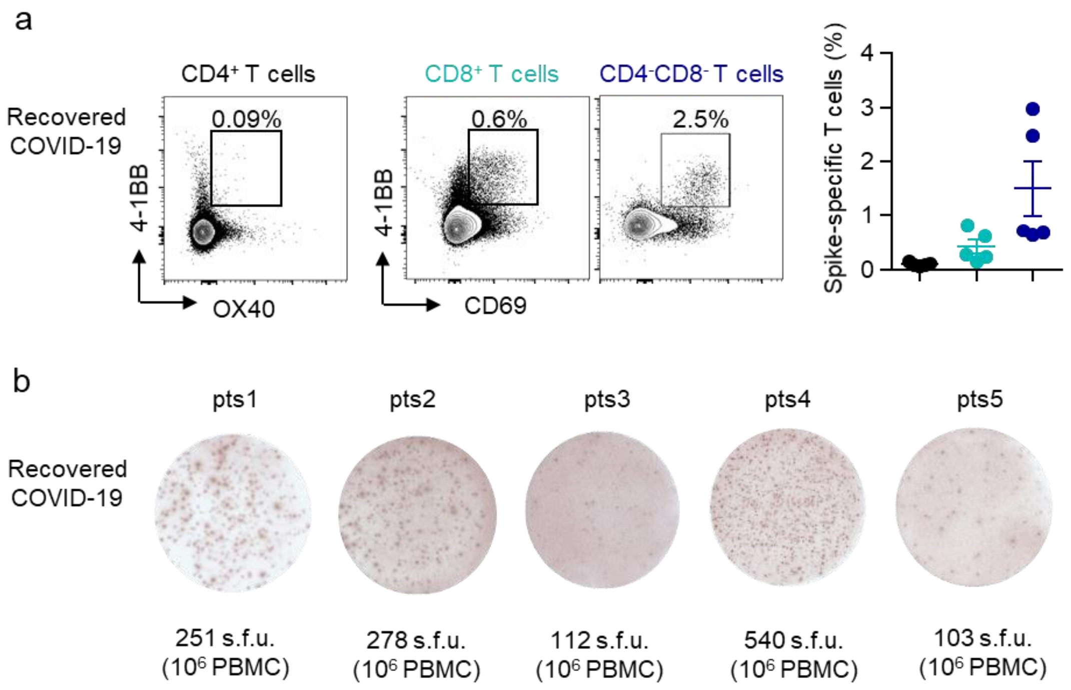

Supplementary Fig. S7 SARS-CoV-2-specific T cells in convalescent COVID-19 patients. (a) PBMCs were stimulated with spike peptide pool (2 μg/ml) for 12 h. SARS-CoV-2-specific $CD4^+$ T cells ($OX40^+$4-$1BB^+$), $CD8^+$ T cells ($CD69^+$4-$1BB^+$) and $CD3^+CD4^-CD8^-$ T cells ($CD69^+$4-$1BB^+$) were measured by flow cytometry. Data were summarized from 5 independent samples. (b) SARS-CoV-2-specific T cells were further detected by IFNγ ELISpot. Images of ELISpot wells of each sample were shown. Spot-forming units (s.f.u) were summarized and adjusted to 10^6 PBMCs.
